# Who gets post-concussion syndrome? An emergency department-based prospective analysis

**DOI:** 10.1186/s12245-014-0031-6

**Published:** 2014-08-20

**Authors:** Latha Ganti, Hussain Khalid, Pratik Shashikant Patel, Yasamin Daneshvar, Aakash N Bodhit, Keith R Peters

**Affiliations:** 1 North Florida South Georgia Veterans Affairs Medical Center, 1601 Archer Road, Gainesville 32610, FL, USA; 2 The University of Florida College of Medicine, Gainesville 32610, FL, USA; 3 Department of Emergency Medicine, UF Health, Gainesville 32610, FL, USA; 4 Department of Radiology, UF Health, Gainesville 32610, FL, USA; 5 Department of Neurological Surgery, UF Health, Gainesville 32610, FL, USA

**Keywords:** Post-concussion syndrome, Mild traumatic brain injury, Emergency department

## Abstract

**Background:**

The objective of this study was to determine who gets post-concussion syndrome (PCS) after mild traumatic brain injury or head injury.

**Methods:**

Patients presented within an hour of mild traumatic brain injury (mTBI). Written informed consent was obtained from all patients, who then provided detailed answers to surveys at the time of injury as well as at 1 week and 1 month follow-up. Statistical analyses were performed using JMP 11.0 for the Macintosh.

**Results:**

The most commonly reported symptoms of PCS at first follow-up were headache (27%), trouble falling asleep (18%), fatigue (17%), difficulty remembering (16%), and dizziness (16%). Furthermore, only 61% of the cohort was driving at 1 week follow-up, compared to 100% prior to the injury.

Linear regression analysis revealed the consumption of alcohol prior to head injury, the mechanism of head injury being a result of motor vehicle collision (MVC) or fall, and the presence of a post-injury headache to be significantly associated with developing PCS at 1 week follow-up, while the occurrence of a seizure post-injury or having an alteration in consciousness post-injury was significantly associated with developing PCS at 1 month follow-up. On multivariate regression analysis, the presence of a headache post-injury was the most robust predictor, retaining statistical significance even after controlling for age, gender, and presence of loss of consciousness (LOC), alteration of consciousness (AOC), post-traumatic amnesia (PTA), seizure, or vomiting.

**Conclusions:**

The results of this prospective study suggest that headache right after the head injury, an alteration of consciousness after the head injury, and alcohol consumption prior to the head injury are significant predictors of developing PCS, which occurs with equal frequency in men and women. Early identification of those who are at risk of developing PCS would diminish the burden of the injury and could potentially reduce the number of missed work and school days.

## Background

Mild traumatic brain injury (mTBI) or concussion is becoming a widespread public health problem [1] and is of especial concern in our youth [2], college athletes [3], and senior citizens [4].

Post-concussion syndrome (PCS) includes a constellation of symptoms that are classified into physical, cognitive, emotional, and sleep problems [5]. Physical problems include headache, nausea, vomiting, balance and visual problems, dizziness, fatigue, sensitivity to light or noise, numbness or tingling, and feeling dazed or stunned. Cognitive problems include feeling mentally ‘foggy,’ speaking slowly, and having difficulty attending, concentrating, executing, judging, processing, remembering, tracking, or understanding. Emotional problems include irritability, sadness, and nervousness. Sleep problems include drowsiness, sleeping more or less than usual, and having trouble falling asleep.

It is not known what causes PCS symptoms to occur and persist or why some people who suffer an mTBI develop PCS while others do not. The objective of this study was to determine whether any initial factors surrounding acute head trauma predict the development of PCS.

## Methods

This was an institutional review board (IRB; The University of Florida Institutional Review Board)-approved prospective observational cohort study of adults presenting to the emergency department (ED) with mTBI, defined as having a Glasgow Coma Score (GCS) of 13 to 15 upon initial presentation to the ED, with head injury having occurred within 24 h of presentation. Written informed consent was obtained from all patients.

The information collected at the time of injury included patient demographics, mechanism of injury, whether alcohol was consumed before the injury, post-symptoms including loss of consciousness (LOC), alteration of consciousness (AOC), seizure, vomiting, headache, or post-traumatic amnesia (PTA), whether retrograde or anterograde (Table 1). An alteration in consciousness was defined as having any of the following: looking or feeling dazed, confusion, difficulty thinking clearly, difficulty responding to mental status questions, inability to describe events immediately before or after the traumatic event, disorientation, or a decreased level of consciousness.

**Table 1:** Association of symptoms in the ED with PCS at 1 week and 1 month follow-up

**Symptom in ED**	**% of whole cohort with this factor**	**PCS at 1 week follow-up**	**PCS at 1 month follow-up**
Loss of consciousness (LOC)	4	NS	NS
Alteration of consciousness (AOC)	45	NS	0.0042
Post-traumatic amnesia (PTA)	35	NS	NS
Headache	57	0.0024	0.0001
Vomiting	7	NS	NS
Seizure	1	NS	0.0520
Mechanism of injury is fall	45	0.0014	NS
Mechanism of injury is MVC	49	0.0107	NS
Prior Hx of TBI	41	NS	NS
Alcohol consumption before the event	53	0.0470	NS

NS, non-significant (*p* value is >0.05).

Patients provided detailed answers to surveys at the time of injury as well as at 1 week and 1 month follow-up. The follow-up was conducted by telephone, via an IRB-approved script, which included the option for patients to decline answering the follow-up surveys. PCS was defined as being present if any of the symptoms listed in Table 2 were present.

**Table 2:** Specific symptoms of PCS at 1 week and 1 month follow-up

	**1 week follow-up (*n*** **= 405)**		**1 month follow-up (*n*** **= 265)**		
	***n***	**% of cohort**	***n***	**% of cohort (*n*** **= 265)**	**% of total cohort (*n*** **= 405)**
Headache	112	27	16	6	4
Nausea	36	9	11	4	3
Vomiting	14	3	6	2	1
Balance problems/dizziness	65	16	26	10	6
Tinnitus	18	4	14	5	3
Sensitivity to light/noise	55	13	12	5	3
Blurred vision/diplopia/flashing lights	41	10	14	5	3
Numbness/tingling	57	14	15		4
Drowsiness	40	10	14	5	3
Fatigue/lethargy	69	17	38	14	9
Sadness/depression	41	10	29	11	7
Nervousness/irritation	54	13	33	12	8
Sleeping more than usual	38	9	24	9	6
Trouble falling asleep	76	18	42	16	10
Feeling ‘slowed down’	52	13	30	11	7
Feeling ‘in a fog’ or dazed	69	8	21	8	5
Difficulty remembering	65	16	40	15	10
Difficulty concentrating	52	13	38	14	9
Driving before?	411	100	267	n/a	66
Driving after?	204	61	176	66	43

n/a, not applicable.

Persons entering the data were blinded to the study hypotheses and outcomes. Data were entered into a secure, web-based application designed to support traditional case report form data capture. Statistical analyses were performed using JMP 11.0 for the Macintosh.

## Results

The cohort consisted of 412 patients, 49% women and 51% men with a median age of 44, IQR 26 to 60, and range 18 to 102 years. Patients presented to the ED within an hour of their head injury (mean 35 min, std dev 21 min) and enrolled upon arrival. The racial composition was 77% white, 18% black, 4% Hispanic, and 1% other (Table 3). Thirty four percent of the cohort was married, 7% were separated or divorced, 6% were widowed, and 52% were single.

**Table 3:** Cohort demographics of those that did and did not have PCS at 1 week follow-up

	**Did have PCS at 1 week follow-up (*n*** **= 213)**	**Did not have PCS at 1 week follow-up (*n*** **= 192)**	***p*** **value**
Age	Median = 43	Median = 47	NS
	IQR = 26 to 57	IQR = 26 to 65	
	Range = 18 to 97	Range = 18 to 102	
Sex	Male = 52%	Male = 51%	NS
	Female = 48%	Female = 49%	
Race	White = 77%	White = 76%	NS
	Black = 18%	Black = 19%	
	Hispanic = 3%	Hispanic = 3%	
	Asian = 1%	Asian = 2%	
	Other = 1%		
Marital status	Married = 38%	Married = 31%	NS
	Separated or divorced = 7%	Separated or divorced = 6%	
	Widowed = 4%	Widowed = 9%	
	Single = 50%	Single = 54%	
Smoker	52%	47%	NS
Arrival by EMS	73%	69%	NS
Mechanism of injury	38% fall	51% fall	0.0085
	55% MVC	43% MVC	0.0159
Alcohol before injury	58%	48%	0.044

NS, non-significant.

Motor vehicular collisions accounted for 45% of mTBI, with another 49% being falls, 5% being assault, and 1% other. Almost three quarters of the cohort (71%) came via emergency medical service/ambulance (EMS), while 24% arrived via private vehicle, 4% arrived by air (due to transfer from another facility), and 1% walked in. For those that arrived via EMS, the prehospital Glasgow Coma Score (GCS) was 13 in 2%, 14 in 8%, and 15 in 90%.

**Figure 1:**
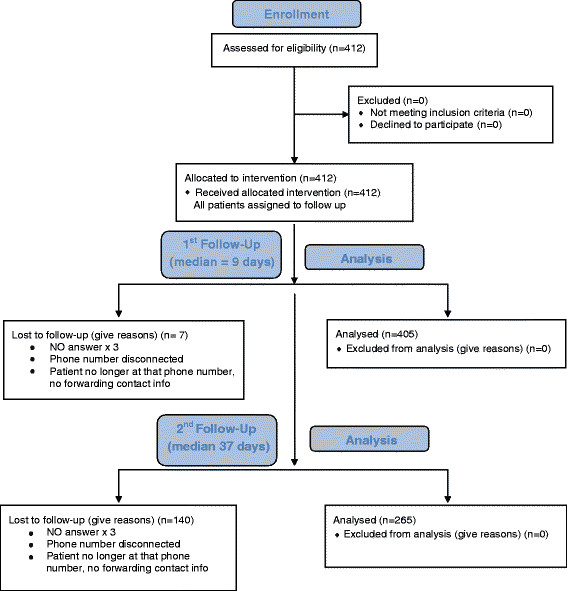
CONSORT flow diagram.

**Figure 2:**
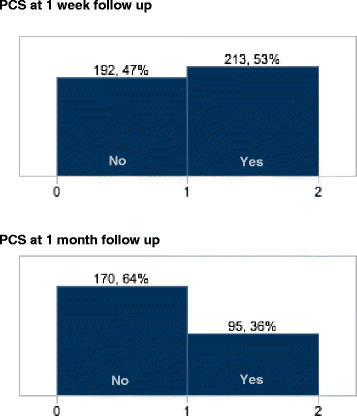
Frequency of post-concussion syndrome (PCS) at 1 week and 1 month follow-up.

A total of 53% reported symptoms consistent with PCS at 1 week (median = 9 days) follow-up. This number decreased to 36% by 1 month (median 37 days) follow-up (Figures 1 and 2). PCS at each follow-up was reported in equal numbers in men and women. The most commonly reported symptoms of PCS at first follow-up were headache (27%), trouble falling asleep (18%), fatigue (17%), difficulty remembering (16%), and dizziness (16%). Sixty one percent of the cohort was driving at 1 week follow-up, compared to 100% prior to the injury.

Alcohol was consumed by 45% of the cohort; 15% consumed within 0 to 6 h of head injury, 21% consumed within 6 to 24 h of the injury, and the remaining 64% consumed alcohol more than 24 h prior to their head injury. Consumption of alcohol prior to head injury was significantly associated with having PCS on 1 week follow-up (*p* = 0.0470).

Linear regression analysis revealed the consumption of alcohol prior to head injury, the mechanism of head injury being a result of motor vehicle collision (MVC) or fall, and the presence of a post-injury headache to be significantly associated with developing PCS at 1 week follow-up, while the occurrence of a seizure post-injury or having an alteration in consciousness post-injury was significantly associated with developing PCS at 1 month follow-up.

On multivariate regression analysis, the presence of a headache post-injury or an alteration in consciousness was significantly associated with developing PCS at 1 week and 1 month, respectively, even after controlling for age and gender. When all symptoms (LOC, AOC, PTA, seizure, vomiting, and headache) were included in the model, only headache retained statistical significance, at both 1 week and 1 month follow-up.

## Discussion

### Prevalence of PCS

Previous studies have reported that between 15% and 30% of mTBI patients meet the criteria for PCS at 1 month [6] and 3 months [7],[8]. Other studies have estimated that up to 50% [9] and even up to 80% [10] of mTBI patients meet the criteria for PCS after 3 months. One study [11] demonstrated 43% of mTBI patients meet the criteria for PCS around 5 days post-injury. The 36% of mTBI patients developing PCS symptoms at 1 month follow-up are in line with these studies, while the 53% reported at 1 week follow-up are higher. Likely, this higher percentage reflects the natural history of head injury, where symptoms are worse early on, with many resolving by 1 month. Nonetheless, the study underscores that more than one third of head-injured patients are still symptomatic 1 month post-injury.

Similar to other studies, the present study used a derivative of the Rivermead Post-Concussion Symptoms Questionnaire to determine whether patients met the criteria for PCS [6],[7],[9],[10],[12]. Some studies have utilized different frameworks for assessing PCS such as the ImPACT Post-Concussion Symptom Inventory [8]. Differences in evaluation measures and sampling criteria may affect the percentages of mTBI patients developing PCS determined by previous studies. The lack of a ‘gold standard’ for distinguishing mild from severe PCS has also been noted [12]. It is possible that percentages of mTBI patients at risk for PCS differ based on the severity of the PCS.

### Comparison to other studies

The present study supports previous findings that have shown no association between gender and PCS post-injury [6],[7],[12]. Ponsford et al. [8] demonstrated an association between the female gender and PCS at 1 week post-injury but not at 3 months post-injury. Reported correlations between the female gender and PCS by previous studies may have failed to distinguish greater report of symptoms by females from greater experience of symptoms [9].

Little research has been conducted concerning the effects of mTBI on driving. The present study suggests that a large amount of the study cohort felt significantly impaired as to refrain from driving. Previous studies have shown that people who experience an mTBI often change their driving habits [13]. Schanke et al. [14] have shown that 6 to 9 years post-mTBI, study participants did not change their driving patterns or driving distance but experienced twice as many motor vehicle accidents as the general population. More research on the effect of mTBI on driving is warranted - particularly when it is safe for mTBI patients to return to driving.

The present study supports post-injury headache as predictive of PCS [6],[7],[9],[10],[15]. Faux et al. [9] demonstrated that ‘immediate verbal recall and quantitative recording of headache’ alone can predict PCS with low to moderate specificity and sensitivity. The literature is divided concerning the correlation between age and PCS. Previous studies have suggested that age is not a risk factor for PCS [6],[7],[11],[15], while others have suggested older age to be a risk factor for PCS [8],[10],[16]. The present study does not strongly suggest age to be predictive of PCS, but the identification of younger age rather than older age as a univariate correlate is worth noting.

The lack of association between PTA and PCS noted in the current study is supported by the literature. One study [17] previously noted inconclusive reports on the association between PTA and PCS; however, recent studies are more conclusive and suggest that PTA is not a risk factor for PCS. Upon a rigorous review of the literature by Carroll et al. [16] as representatives of a task force on mTBI, no associations between duration of PTA and slower recovery or duration of PTA and persistence of symptoms after mTBI were found. Other recent studies have also suggested no association between PTA and PCS [7],[8],[11],[15],[16],[18]. Savola et al. [6] found PTA to be a univariate correlate of PCS but not a multivariate correlate.

Mild TBI is caused by any blow or jolt to the head that disrupts normal brain functioning. There is a significant difference in mTBI populations that report to different emergency departments. The present study's cohort experienced injuries mainly from motor vehicular collisions and falls. Ponsford et al. [15] had a high percentage of mTBI patients who sustained sporting injuries. The diverse mechanisms of mTBI affect results and add another variable when comparing studies examining mTBI patients. They noted that most mTBI patients who suffered problems post-injury were injured in motor vehicular accidents. There were also a greater number of females injured in motor vehicular accidents in comparison to a greater number of males injured in sporting accidents. The sporting accident population suffered fewer problems than the motor vehicular accident population, and at surface value, the data indicated a correlation between female gender and PCS when there may have just been a relation between mechanism of injury and PCS [15]. Future research should examine the relationship between mechanism of mTBI and PCS and what aspects of specific mechanisms contribute to poorer outcomes.

Many prospective observational cohort studies have used multivariate analyses to identify predictors of PCS [6],[7],[12],[19]. Factors that are not commonplace such as ‘all or nothing coping behavior’ [7] have been proposed as predictors of PCS. Some of these factors are not ordinarily monitored or studied in the literature, and confirmatory studies are therefore not performed. As a result, there is an amalgam of factors predictive of PCS that have been independently identified but have not appreciably contributed to the literature regarding PCS and are difficult to critically examine. Carroll et al. [16] analyzed and synthesized the literature regarding mTBI prognosis and identified very few factors that are predictive of PCS - the ones that are predictive of PCS, such as litigation, have nothing to do with mTBI. Meares et al. [11] compared an mTBI population to a control population and suggested that mTBI does not predict acute PCS. The overlap of PCS symptoms with other disorders such as depression and post-traumatic stress disorder has been noted, and there has been a discussion regarding the biological and psychological underpinnings of PCS [17],[20],[21]. Given the considerable ambiguity, inconsistency, and lack of uniformity surrounding the literature regarding PCS, it is difficult to meaningfully contribute to an understanding of the etiology of PCS. Regardless, the fact that certain populations of mTBI patients have poorer outcomes than others is indisputable, and research concerning why this is the case needs to be performed. Researchers should be cognizant of using study methods that are reproducible, focus on predictive factors or outcomes that have utility in medical practice, and attempt to build upon previous research that has been performed.

### Strengths and weaknesses

The biggest strengths of the current study include the following: (1) recruitment of patients in the hyperacute phase following injury (within 1 h), (2) its prospective design, (3) emergency department setting, and (4) large cohort size with an equal number of men and women.

The limitations of this study include the following: (1) this was a single-center study - our emergency department is a level 1 trauma center in a college town which may have skewed the population. Results may have been different in a community emergency department setting or one in which the demographic composition was different, for example. (2) Because follow-up relied on self-report surveys, accuracy of reporting symptoms, potential secondary gain in positive reporting, and variability in interpretation of symptoms between study participants must be factored into interpretation of the present study's findings.

## Conclusions

The results of this prospective study suggest that headache right after the head injury, an alteration of consciousness after the head injury, and alcohol consumption are significant predictors of developing PCS, which occurs with equal frequency in men and women. Early identification of those who are at risk of developing PCS would diminish the burden of the injury and could potentially reduce the number of missed work and school days.

## Competing interests

The authors declare that they have no competing interests.

## Authors' contributions

LG, ANB, PSP, and YD conceived the study. LG, ANB, PSP, YD, and HK collected the data. ANB, HK, and LG performed the statistical analyses, and PSP and YD cross-checked the data. LG and KRP supervised the conduct of the research and data collection. LG and HK drafted the manuscript, and all authors contributed substantially to its revision. All authors read and approved the final manuscript.
